# Metabolomic Insights into the Nutritional Status of Adults and Adolescents with Phenylketonuria Consuming a Low-Phenylalanine Diet in Combination with Amino Acid and Glycomacropeptide Medical Foods

**DOI:** 10.1155/2017/6859820

**Published:** 2017-12-31

**Authors:** Bridget M. Stroup, Denise M. Ney, Sangita G. Murali, Frances Rohr, Sally T. Gleason, Sandra C. van Calcar, Harvey L. Levy

**Affiliations:** ^1^Department of Nutritional Sciences, University of Wisconsin-Madison, Madison, WI, USA; ^2^Boston Children's Hospital, Harvard Medical School, Boston, MA, USA; ^3^Department of Molecular and Medical Genetics, School of Medicine, Oregon Health and Science University, Portland, OR, USA

## Abstract

**Background:**

Nutrient status in phenylketonuria (PKU) requires surveillance due to the restrictive low-Phe diet in combination with amino acid medical foods (AA-MF) or glycomacropeptide medical foods (GMP-MF). Micronutrient profiles of medical foods are diverse, and optimal micronutrient supplementation in PKU has not been established.

**Methods:**

In a crossover design, 30 participants with PKU were randomized to consume AA-MF and Glytactin™ GMP-MF in combination with a low-Phe diet for 3 weeks each. Fasting venipunctures, medical food logs, and 3-day food records were obtained. Metabolomic analyses were completed in plasma and urine by Metabolon, Inc.

**Results:**

The low-Phe diets in combination with AA-MF and GMP-MF were generally adequate based on Dietary Reference Intakes, clinical measures, and metabolomics. Without micronutrient supplementation of medical foods, >70% of participants would have inadequate intakes for 11 micronutrients. Despite micronutrient supplementation of medical foods, inadequate intakes of potassium in 93% of participants and choline in >40% and excessive intakes of sodium in >63% of participants and folic acid in >27% were observed. Sugar intake was excessive and provided 27% of energy.

**Conclusions:**

Nutrient status was similar with AA-MF and Glytactin GMP-MF. More research related to micronutrient supplementation of medical foods for the management of PKU is needed.

## 1. Introduction

Phenylketonuria (Online Mendelian Inheritance in Man 261600) is an inherited metabolic disease that is characterized by a loss of function of the hepatic phenylalanine hydroxylase (PAH^8^; Enzyme Commission number 1.14.16.1), thereby limiting the hydroxylation of phenylalanine to tyrosine [1]. Diagnosis and initiation of treatment are required to prevent severe cognitive impairment caused by toxic accumulations of Phe in the brain [2, 3]. Nutritional management requires compliance with a lifelong low-Phe diet, which is central to the treatment of PKU. The low-Phe diet consists of a controlled amount of natural protein to provide minimum Phe requirements and consumption of elemental amino acid medical foods (AA-MF) or glycomacropeptide medical foods (GMP-MF), distributed three times per day, to provide the majority of dietary protein needs [4].

Glycomacropeptide is a 64-amino acid glycophosphopeptide (7–11 kDa) and is part of the κ-casein micelle in bovine milk that is cleaved by chymosin during the cheese-making process [5]. GMP is not a complete protein and requires supplementation with the following indispensable amino acids: Arg, His, Leu, Trp, and Tyr [6, 7]. Our randomized, controlled crossover trial demonstrated that Glytactin GMP-MF are a safe and acceptable alternative with fewer reported gastrointestinal side effects compared to AA-MF, which may improve lifelong medical food compliance [8]. This evidence supports the new paradigm for use of primarily intact protein from GMP-MF for the nutritional management of PKU [4, 9].

The focus on dietary macro- and micronutrient patterns has progressed from deficiency prevention towards an emphasis on disease prevention and wellness. Maintenance of adequate nutritional status, based on dietary intake and biomarkers, is challenging for PKU due to the small amount of natural protein that can be incorporated into the low-Phe diet [10]. To accommodate the restrictive low-Phe diet, PKU medical foods have been supplemented with chemically derived vitamins and minerals within the last 25 years, and more recently, with essential fatty acids [10, 11]. The bioavailability of both natural and chemically derived vitamins and minerals is complex and impacted by dietary constituents, such as fiber and fat content [12–15]. For example, there is evidence of increased bioavailability of chemically derived folic acid and retinyl palmitate (vitamin A) and reduced bioavailability of sodium selenite, a common chemically derived form of selenium [12, 16, 17].

Research groups have reported clinical data from PKU subjects indicating low or deficient levels of several micronutrients, including iron, selenium, vitamin B-12, zinc, and fatty acids, such as total *n*-3 fatty acids, docosahexaenoic acid (DHA), and eicosapentaenoic acid (EPA) [10, 11, 18–20]. This finding is likely due, in part, to reduced bioavailability of synthetic nutrients, variable content of nutrients in medical foods, or low medical food compliance. At the same time, there is evidence of high serum concentrations of folate and selenium [18]. Regardless, prior studies provide nutritional status assessment data based on mostly children with PKU using AA-MF. Pinto et al. recently reported similar concentrations of Phe, select micronutrients (iron, vitamins D and B-12, folic acid, and zinc), and lipoproteins in individuals with PKU consuming a combination of AA-MF and Glytactin GMP-MF; micronutrient intake was not reported [21]. The objective of this study was to utilize metabolomics and traditional dietary evaluation methods to assess the nutritional status of adults and adolescents with PKU consuming AA-MF and Glytactin GMP-MF in combination with a low-Phe diet. Our comprehensive approach assessed dietary intake of macronutrients and micronutrients from medical foods as well as natural foods and modified low-protein foods.

## 2. Methods

### 2.1. Study Design and Protocol

**Figure 1:**
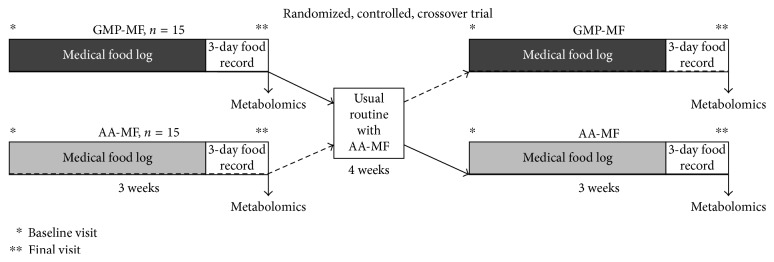
Experimental design. AA-MF, amino acid medical foods; GMP-MF, glycomacropeptide medical foods.

Assessment of macro- and micronutrient intake of adults with PKU consuming a low-Phe diet in combination with AA-MF compared to Glytactin GMP-MF was an important secondary aim of our randomized, controlled, crossover clinical trial [8]. Participants completed the study protocol either at the Waisman Center (*n* = 19) or at Boston Children's Hospital (*n* = 11) [8]. The University of Wisconsin-Madison Health Sciences Institutional Review Board approved the study protocol. All participants provided written informed consent. The trial was registered at ClinicalTrials.gov as NCT01428258. As previously reported, inclusion criteria included a diagnosis of PKU that was early treated with medical food and a current prescription providing greater than 50% of daily protein needs from medical foods [8]. The study protocol included baseline (day 1) and final (day 22) study visits for both AA-MF and GMP-MF treatments, where fasting blood samples and 3-day food records were obtained (Figure 1) [8].

Participants consumed a low-Phe diet and were randomized to consume AA-MF and GMP-MF for 3 weeks each. The main change in the GMP-MF treatment was the substitution of all protein equivalent (PE) intake from AA-MF with GMP-MF. Participants consumed their usual Phe-free AA-MF, as prescribed by their home metabolic clinics, which resulted in the use of 15 different AA-MF [8]. The list of AA- and GMP-MF used by participants has been previously reported [8]. Cambrooke Therapeutics donated the GMP-MF from 2010 to 2015, which contained Glytactin, a patented formulation of ∼70% glycomacropeptide (cGMP-20, Arla Foods Ingredients) and ∼30% supplemental AAs (Arg, His, Leu, Trp, and Tyr). Consecutive 3-day food records and fasting venipunctures were collected prior to the start and the end of both dietary treatments. A daily medical food log was kept by subjects for each of the 3-week treatments and used to assess intake of PE from medical foods compared with prescribed intake of PE from medical foods (average medical food prescription in study: 0.85 ± 0.03 g PE/kg/d). Median intake of PE from medical food was not different between AA-MF and GMP-MF (medians, g PE/kg/d: AA-MF, 0.78; GMP-MF, 0.76; *p*=0.94) and indicates that participants obtained approximately 90% of the average medical food prescription. Compliance with AA-MF and GMP-MF was previously reported [8].

### 2.2. Assessment of Medical Food and Natural Food Intake

Nutrient intake distributions (10th, median, and 90th percentiles) for macro- and micronutrients from the whole diet, medical foods, and natural foods, based on 3-day food records at the end of AA-MF and GMP-MF treatments, are reported for the first time. Food record analyses were conducted by a registered dietitian (RD) experienced in standardized diet entry using Food Processor SQL (version 10.12.0; ESHA) and were surveilled by a second RD for quality assurance. Nutrient intake of participants <18 y was combined with that of adult participants due to similar PE intake from MF with both treatments (means ± SE, adults, 54 ± 3 g PE from MF, *n* = 25; minors, 47 ± 6 g PE from MF, *n* = 5; *p*=0.38) and the small sample size. Natural foods were defined as all food and beverages that were not medical foods intended for the treatment of PKU. Intake of modified low-protein foods (MLPF) was investigated, and MLPF were defined as foods that were “modified to be low in protein and formulated for oral consumption for individuals for whom a condition or disorder prevents typical food consumption. This does not include foods that are naturally low in protein, such as some fruits or vegetables” [22]. Because MLPF are intended to add variety to the low-Phe PKU diet, MLPF were categorized as “natural food.”

### 2.3. Assessment of Inadequate and Excessive Intake of Macro- and Micronutrients

To investigate inadequate and excessive intakes of macronutrients and micronutrients, we compared the average 3-day intake of each nutrient for AA- and GMP-MF at the end of the 3-week treatment period against the gender- and age-appropriate nutrient reference cutoffs for each individual participant. The United States' acceptable macronutrient distribution range (AMDR) was used to evaluate percent caloric intake from total protein, carbohydrate, and fat, and the Dietary Guidelines for Americans 2015–2020 (DGA) were used to evaluate percent caloric intake from sugar and saturated fat [23, 24]. The United States' estimated average requirements (EAR) and adequate intake (AI) were used as reference cutoffs to evaluate inadequate total micronutrient intakes, and the tolerable upper intake levels (UL) were used as reference cutoffs to evaluate excessive total micronutrient intakes [25, 26]. Excessive intakes of vitamin A, vitamin E, niacin, folate, and magnesium from medical foods, as opposed to the whole diet, were evaluated because the UL for those micronutrients represent intakes from pharmacological sources and not natural foods [25].

To estimate the percentage of participants with inadequate or excessive intakes of vitamins A and E, assumptions related to vitamin form and the conversions used to account for bioavailability were made. For vitamin A, we reported intake using international units (IUs) rather than retinol activity equivalents (RAE) because American food labels generally report vitamin A content of foods as percent of daily value or IU and do not account for differences related to bioavailability of preformed vitamin A (retinol) and provitamin A carotenoids (*α*-carotene, *β*-carotene, and *β*-cryptoxanthin). To utilize the EAR and UL to evaluate inadequate and excessive intakes of vitamin A, vitamin A intake from medical foods was evaluated as retinol, given that vitamin A is added to medical foods as retinyl palmitate or retinyl acetate and vitamin A intake from natural foods was evaluated as *β*-carotene. We evaluated vitamin A intake from natural foods as *β*-carotene because our participants had low intakes of fortified grain products, and many of the low-Phe fruits and vegetables consumed by our participants generally have high contents of *β*-carotene. To evaluate inadequate and excessive intake of vitamin E, vitamin E from medical foods was evaluated as dl-alpha tocopherol, as this is the most common form of vitamin E added to medical foods. Vitamin E from natural foods was evaluated as l-alpha-tocopherol to account for differences in bioavailability.

### 2.4. Clinical Measurements

Nontargeted metabolomic analyses on fasting plasma samples from participants with PKU (*n* = 10) compared to fasting plasma samples from a gender- and age-matched control population (*n* = 15) and on aliquots from 24-hour urine collections from participants with PKU (*n* = 9) were carried out by Metabolon, Inc. (Durham, NC, USA). The plasma and urine samples used for the metabolomic analyses were obtained from a subset of the 30 participants enrolled in the previously reported clinical trial [8]. Methods for metabolomic analyses on plasma and urine samples have been previously reported [27]. Hemoglobin, plasma ferritin, and serum zinc were analyzed using standard techniques at the clinical laboratories located at the University of Wisconsin Hospital and Clinics and Boston Children's Hospital. Serum methylmalonic acid (MMA) concentrations were analyzed using gas chromatography/mass spectrometry by Metabolite Laboratories, Inc. (Denver, CO, USA).

### 2.5. Statistical Analysis

All statistical analyses were performed using SAS version 9.4, and assumptions of normality and equal variance were tested. Most analyses used PROC MIXED (SAS Institute Inc.). Subject characteristics and DXA scan data were analyzed using ANOVA with effects for sex and genotype (classical or variant PKU). Nutrient and MLPF intake data were analyzed using ANOVA with effects for diet (AA-MF or GMP-MF), genotype, and diet by genotype interactions. Two analyses were conducted for micronutrient intakes from medical foods using ANOVA with effects for diet, genotype, and diet by genotype interactions. The first analysis included all 30 subjects, while the second analysis only included subjects that consumed micronutrients from both AA-MF and GMP-MF (*n* = 21–27). The Kruskal–Wallis test was used to test for differences due to diet or genotype, if data were skewed. Statistical analyses for the metabolomics were performed in Array Studio version 7.2 and R version 3.02. The metabolites in the metabolomic analyses for both plasma and urine samples were rescaled to set the median to one, based on all samples. Statistical analyses were conducted to detect differences in fold change. To detect differences in the metabolomics of the plasma samples, paired *t*-tests were used to compare differences between treatments and PKU genotypes. Welch's two-sample *t*-tests were used to detect differences between a dietary treatment and a PKU genotype against a control population. To detect differences in the metabolomics of the urine samples, paired *t*-tests were used to detect differences between treatments and genotypes, and ANOVA was used to test for treatment, genotype, and treatment by genotype interactions. Data from urine samples were corrected for osmolality prior to statistical analysis; the dietary treatments did not have a significant effect on urine osmolality. Statistical significance was set at *p* < 0.05.

## 3. Results

### 3.1. Participants

Thirty participants were enrolled in the clinical trial (18 females and 12 males) and included 25 adults (18–49 y) and 5 minors (15–17 y) [8]. Participant characteristics are summarized in Table 1. Of the 20 participants categorized with classical PKU, defined as having a PAH genotype and/or inadequate response to sapropterin dihydrochloride resulting in a severe PKU phenotype, eight were male, ten were female, and two were minors. Of the 10 participants categorized with variant PKU, defined as having a mild PAH genotype and/or response to sapropterin dihydrochloride resulting in a mild PKU phenotype, two were male, five were female, and three were minors. Five participants used sapropterin hydrsapropterin dihydrochloride consistently throughout the study.

### 3.2. Macronutrient Intake Profile of the Whole Diets

**Figure 2:**
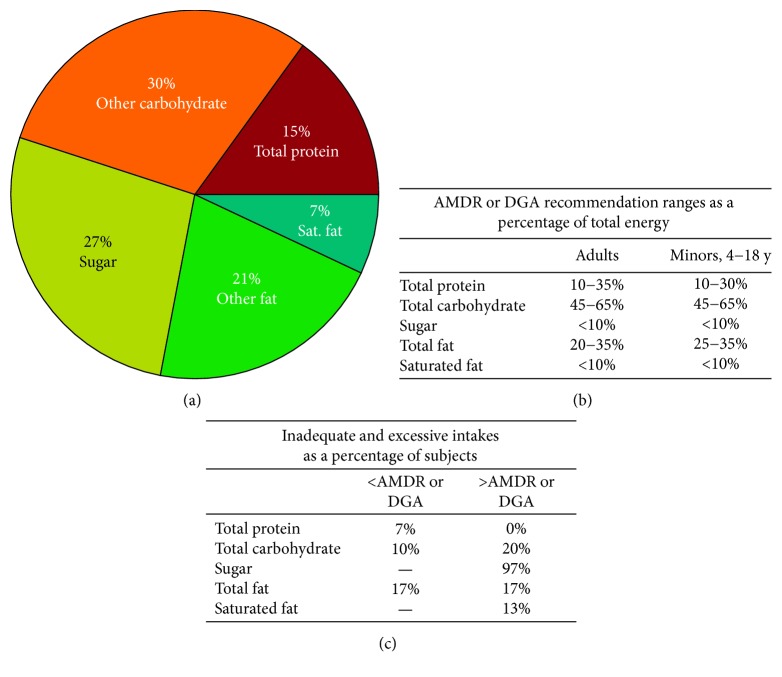
Caloric contribution from carbohydrate, sugar, protein, fat, and saturated fat was calculated for the low-Phe diet in combination with AA-MF as a percentage of total energy intake (a). Macronutrient distribution was similar for GMP-MF (data not shown). Caloric contribution from macronutrients was compared to the age-appropriate AMDR (protein, carbohydrate, and fat) or the DGA (sugar and saturated fat) (b) for each participant to determine the percentage of subjects that were below or above the AMDR or DGA (c). Percentage of participants with inadequate and excessive macronutrient distributions were similar for GMP-MF (data not shown). AA-MF, amino acid medical food; AMDR, acceptable macronutrient distribution ranges; DGA, Dietary Guidelines for Americans; GMP-MF, glycomacropeptide medical foods; Sat. fat, saturated fat.

The low-Phe diets in combination with AA-MF and GMP-MF were generally constant with similar total intakes (i.e., the whole diet) of energy, protein, carbohydrate, fat, Phe, and PE from medical foods (medians, 53–59 g PE/d) (Table 2). Percent calories from total protein, carbohydrate, sugar, fat, and saturated fat as a percentage of total energy intake were similar for both AA-MF and GMP-MF whole diets and are reported only for the low-Phe diet in combination with AA-MF (Figure 2). However, intakes of carbohydrate, fat, and saturated fat from medical foods were significantly higher with GMP-MF. The significantly higher carbohydrate intake with GMP-MF was driven by the higher sugar intake with GMP-MF (medians, 53 g/d sugar with GMP-MF compared to 36 g/d sugar with AA-MF; *p*=0.10). Participants with variant PKU consumed more protein and Phe from natural foods and a higher percentage of total energy intake from fat and saturated fat compared with participants with classical PKU [28]. Additionally, significant differences were found in the intake of 15 of 18 dietary amino acids, which have been previously reported [29].

### 3.3. Low Intake of Modified Low-Protein Foods among Participants

Of 30 participants, only 13 consumed MLPF with both AA- and GMP-MF treatments. Macronutrient and Phe intake from MLPF was similar with both diets (Table 3). Interestingly, only 211–257 kcal (medians), <1 g of protein, and 51–62 g carbohydrates from MLPF were consumed, which comprised ∼10% of median total calories and ∼17% of median total carbohydrate intake with both diets (Table 3). Types of MLPF consumed most often during this study included low-protein baking mixes, pasta, cereal, and cheese. Given that most of the MLPF consumed during this study were comprised of mainly starch, micronutrients are not reported. The surprising low rates of MLPF intake and low caloric contribution to the low-Phe diet are likely two-fold: (1) differences in public and private coverage of MLPF in the states of the home metabolic clinics within our heterogeneous participant population and (2) the majority of MLPF to which participants had access required some preparation and may have acted as a barrier to excessive consumption. To the best of our knowledge, this study is the first to report macronutrient intake patterns from MLPF of individuals with PKU in the United States.

### 3.4. Micronutrient Intake Profile of the Diets

Vitamin and mineral intake profiles of the low-Phe diets in combination with AA-MF and GMP-MF are summarized in Tables 4 and 5. The greater intake of natural foods in the diet of participants with variant PKU was associated with greater intakes of vitamin D, vitamin B-12, iodine, selenium, and zinc compared to subjects with classical PKU [28]. Dietary intakes of 17 of 25 vitamins and minerals from the whole diet and from medical foods were similar between the AA-MF and GMP-MF treatments. Dietary intakes from natural foods were not different between AA-MF and GMP-MF, providing further support that the diets were generally constant except for the type of medical food consumed. Participants consumed significantly more dietary thiamin and copper from the whole diet and medical foods with AA-MF compared to GMP-MF and consumed significantly less dietary choline and sodium from the whole diet and medical foods with AA-MF (Tables 4 and 5).

Because several AA-MF and GMP-MF do not contain micronutrients, subanalyses were conducted to test whether there were differences in dietary intakes of micronutrients from only the medical foods that did contain the micronutrient of interest (*n* = 21–27). Of the subanalyses conducted for the 25 dietary micronutrient intakes from medical foods, only the 3 subanalyses, vitamin E, iron, and zinc, demonstrated different outcomes from the full analysis (*n* = 30). There was no significant difference in dietary intake of zinc from medical food based on the subanalysis (*n* = 21). However, the subanalyses indicated that AA-MF supplemented with vitamins and minerals tended to have higher contents of vitamin E and iron compared to GMP-MF.

### 3.5. Inadequate and Excessive Micronutrient Intakes per the Dietary Reference Intakes

To assess the rates of inadequate micronutrient intakes with AA-MF and GMP-MF treatments, dietary intakes from the whole diet were compared to the EAR or AI, which were evaluated for each individual participant based on age and gender (Tables 4 and 5). There were high rates of inadequate micronutrient intakes from the whole diet, expressed as percentages of participants below the EAR or AI with AA-MF and GMP-MF treatments, for the following micronutrients: potassium (93% for both treatments), choline (>40%), vitamin D (33% for both treatments), and iodine (>23%). Although approximately one-third of participants did not meet the EAR for vitamin D, we have previously shown that our participants with PKU have no evidence of vitamin D deficiency based on concentrations of serum 1,25-dihydroxyvitamin D (means ± SE, pg/mL: AA-MF, 65.4 ± 3.39; GMP-MF, 71.9 ± 4.10; *p*=0.079) and 25-hydroxyvitamin D (means ± SE, ng/mL: AA-MF, 33.6 ± 1.53; GMP-MF, 33.8 ± 1.70; *p*=0.797) [30]. Furthermore, there was some evidence of inadequate micronutrient intakes from the whole diet with AA-MF and GMP-MF treatments for the following micronutrients: magnesium, vitamin E, biotin, zinc, selenium, pantothenate, vitamin K, vitamin C, copper, and thiamin.

In addition to rates of inadequate intakes, micronutrient intakes from either the whole diet or medical foods were compared to the UL to investigate rates of excessive micronutrient intake. There were high rates of excessive micronutrient intakes from medical foods, expressed as percentages of participants above the UL with AA-MF and GMP-MF treatments, for the following micronutrients: magnesium (>37%), folic acid (>27%), and niacin (20% with AA-MF) (Tables 4 and 5). Because the UL for magnesium, folate, and niacin applies to synthetic sources only, comparison of the UL against the intake from the whole diet was not evaluated for those micronutrients. Not surprisingly, >63% of participants had sodium intakes from the whole diet with AA-MF and GMP-MF that were above the UL (>2300 mg/d). Participants consumed significantly more sodium from the whole diet with GMP-MF due to the significantly higher sodium intake from GMP-MF compared to AA-MF (medians, 1140 mg sodium/d from GMP-MF compared to 413 mg sodium/d from AA-MF, *p* < 0.0001, *n* = 30). Nonetheless, the majority of the dietary sodium intake was obtained from *natural foods* (medians, 2041 mg sodium/d with GMP-MF compared to 2206 mg sodium/d with AA-MF, *p*=0.69).

To test whether the vitamin and mineral content of low-Phe diets would be adequate without intake of medical foods, we compared the micronutrient intake from natural foods to the gender- and age-appropriate EAR or AI for each participant. Without medical foods, more than 70% of participants would not meet the EAR or AI for the following micronutrients: choline (100%), potassium (>97%), iodine (>93%), calcium (>90%), vitamin D (>90%), magnesium (>87%), biotin (>83%), pantothenate (>83%), vitamin E (>80%), zinc (>77%), and selenium (>73%). Interestingly, without medical food intake, >90% of participants would be able to obtain vitamin A from natural foods alone to meet or exceed the EAR due to high intakes of provitamin A carotenoids from green leafy vegetables (i.e., spinach and lettuces), squashes, carrots, and tomato products, such tomato-based pasta sauce (Table 4).

### 3.6. Metabolomics

Metabolomic analyses of Met metabolism, vitamins, and food components in plasma and urine samples are reported in Supplemental Tables 1–3.

#### 3.6.1. Vitamins A, C, and E

Plasma retinol concentrations were similar between AA-MF and GMP-MF treatments and 17% higher (*p*=0.17) compared to controls. Given that plasma retinol is homeostatically controlled, the somewhat higher retinol levels in PKU subjects are unexplained. Significantly higher plasma retinal concentrations (∼1.7-fold ↑) were found in participants with variant and classical PKU who consumed AA-MF and GMP-MF compared to a control population (GMP-MF versus controls, *p*=0.0006; AA-MF versus controls, *p*=0.0053). Although plasma retinal is not recognized as a biomarker of vitamin A status, this agrees with the high dietary intakes of preformed vitamin A with both AA-MF and GMP-MF in participants with PKU. Not surprisingly, given the low intake of citrus fruits, pumpkin, and red pepper, participants with variant and classical PKU and participants who consumed AA-MF and GMP-MF showed 0.3-fold lower plasma *β*-cryptoxanthin concentrations (*p*=0.0064) compared to controls [12]. Consistent with similar intakes of dietary vitamin C, there were no differences in urinary ascorbate and dehydroascorbate concentrations.

Plasma *α*-tocopherol concentrations were similar between AA-MF and GMP-MF treatments and between participants consuming AA-MF or GMP-MF compared to controls, despite significantly lower vitamin E intakes with GMP-MF. However, plasma *α*-tocopherol concentrations were 1.25-fold higher in participants with variant PKU compared to classical PKU (*p*=0.03). Furthermore, plasma *α*-tocopherol concentrations were 0.8-fold lower in participants with classical PKU compared to controls (*p*=0.054). Urinary excretion of alpha-CEHC (2,5,7,8-tetramethyl-2-(2′-carboxyethyl)-6-hydroxychroman), a major water-soluble *α*-tocopherol metabolite, was not significantly different based on dietary treatment or PKU genotype. Interestingly, plasma concentrations of gamma-CEHC (3-(2,7,8-trimethyl-3,4-dihydro-2H-chromen-2-yl) propanoate), a water-soluble *γ*-tocopherol metabolite, were approximately twofold higher in participants consuming GMP-MF and AA-MF (GMP-MF versus controls, *p*=0.03; AA-MF versus controls, *p*=0.07) and participants with variant PKU compared to the control population (Variant PKU versus controls, *p*=0.002). Urinary excretion of gamma-CEHC concentrations was 2.8-fold higher with GMP-MF compared to AA-MF (*p*=0.028). This is the first study to report dietary intake of vitamin E and metabolites for both *α*- and *γ*-tocopherol in plasma and urine.

#### 3.6.2. B-Vitamins

Compared to AA-MF, 0.48-fold less thiamin was excreted in the urine with GMP-MF (*p*=0.043), which agrees with the significantly lower dietary thiamin intake with GMP-MF. No significant treatment differences were found in urinary riboflavin concentrations, consistent with similar dietary riboflavin intakes. Higher plasma nicotinamide concentrations were observed in participants consuming AA-MF (1.42-fold ↑, *p*=0.07) and GMP-MF (1.47-fold ↑, *p*=0.06) compared to a control population. Plasma nicotinamide concentrations that were 1.66-fold higher were observed in participants with variant PKU compared to controls (*p*=0.01). These results align with similar dietary niacin intakes between treatments and the observation that 20% of participants exceeded the UL for dietary niacin intake with AA-MF.

Despite similar dietary pantothenate intakes and urinary pantothenate concentrations, plasma pantothenate concentrations in participants with classical and variant PKU and participants who used GMP-MF were significantly higher compared to controls (classical PKU versus controls, 1.34-fold ↑, *p*=0.02; variant PKU versus controls, 1.58-fold ↑, *p*=0.03; GMP-MF versus controls, 1.48-fold ↑, *p*=0.01). In agreement with similar dietary vitamin B-6 intakes between treatments and PKU genotype, no significant differences related to diet or genotype were found in plasma concentrations of pyridoxal and pyridoxate or urine concentrations of pyridoxal, pyridoxate, and pyridoxamine. Additionally, plasma levels of pyridoxal and pyridoxate in PKU subjects were not different compared with controls.

Although >40% of participants had dietary choline intakes below the EAR and obtained higher intakes of choline with GMP-MF compared to AA-MF, there were no significant differences in plasma choline concentrations among our participants related to diet or genotype and in comparison with the control population. However, this finding is not surprising, considering that plasma choline is homeostatically controlled [31]. No differences in urinary excretion of choline-related metabolites, dimethylglycine and betaine, between dietary treatments nor PKU genotype were found. However, participants with variant PKU had 1.46-fold higher plasma dimethylglycine compared to classical PKU (*p*=0.013), and participants with classical PKU had 0.8-fold lower plasma betaine concentrations compared to controls (*p*=0.042). Taken together, choline may be a nutrient of concern in PKU.

#### 3.6.3. Taurine

**Figure 3:**
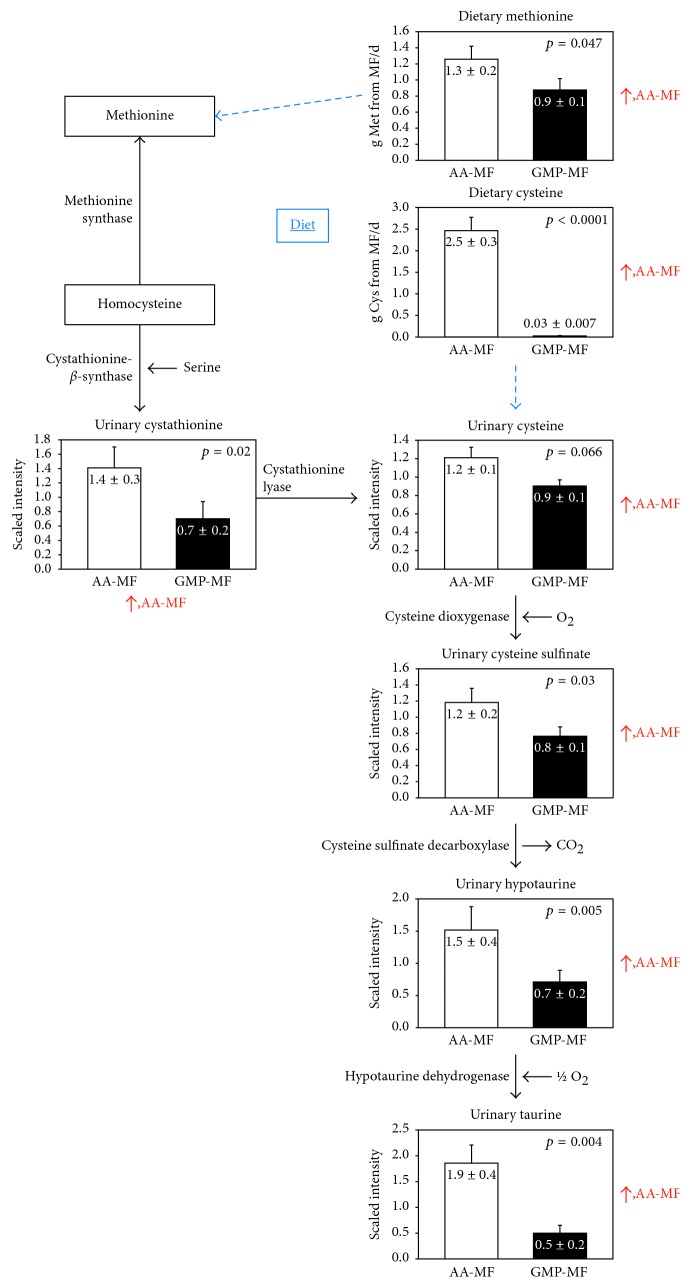
Urine metabolomics of sulfur-containing amino acid (Met and Cys) metabolism, *n* = 9. Values are means ± SE. Participants with PKU consumed significantly more Met and Cys from medical foods with AA-MF compared to GMP-MF, *n* = 8 (means ± SE; Met, g/d: AA-MF, 1.3 ± 0.2, versus GMP-MF, 0.9 ± 0.1, *p*=0.047; Cys, g/d: AA-MF, 2.5 ± 0.3, versus GMP-MF, 0.3 ± 0.007, *p* < 0.0001). Participants had higher urinary excretion of taurine and taurine-related metabolites (hypotaurine, cysteine sulfonate, cysteine, and cystathionine), possibly related to higher intakes of sulfur-containing amino acids and increased need to excrete sulfur. AA-MF, amino acid medical foods; GMP-MF, glycomacropeptide medical foods.

Interestingly, despite similar dietary taurine intakes and plasma taurine concentrations, 0.2-fold less taurine was excreted in the urine with GMP-MF compared to AA-MF (*p*=0.002). Furthermore, urinary excretion of taurine-related metabolites (hypotaurine, cysteine sulfinate, cysteine, and cystathionine) was significantly higher with AA-MF compared to GMP-MF (Figure 3). We have previously reported high urinary sulfate excretion with AA-MF (that also exceeded the reference range) compared to GMP-MF (urinary sulfate excretion (mEq/d, means ± SE); AA-MF, 34 ± 3, versus GMP-MF, 12 ± 3, *p*=0.0008) [30]. Current sulfur-containing amino acid content of AA-MF provides ∼3 times the World Health Organization's recommendation for daily intakes of Met and Cys [32]. Moreover, higher urinary excretion of sulfate, taurine, and taurine-related metabolites with AA-MF may be related to increased need to excrete sulfur with higher intake of sulfur-containing amino acids, Met and Cys, from AA-MF compared with GMP-MF [33]. Taken together, these data suggest that the sulfur-containing amino acid content of AA-MF might be higher than what is needed to support protein synthesis and one-carbon metabolism.

#### 3.6.4. Sweeteners and Inositol

The artificial sweetener acesulfame was added to both AA-MF and GMP-MF, and consumption was higher in PKU subjects as reflected in threefold higher plasma levels of acesulfame in PKU subjects compared to controls (scaled intensity means, variant, 0.76; classical, 0.63; controls, 0.237; *p*=0.067). Notably, urinary acesulfame excretion was 6 times higher with GMP-MF compared to AA-MF (*p*=0.005). Plasma erythritol and urinary erythritol excretion were dramatically higher with GMP-MF compared to AA-MF (*p*=0.019to0.003); plasma levels were also higher with GMP-MF compared with controls (*p*=0.006). Erythritol is a partially absorbed sugar alcohol that is approved by the FDA for use as a food additive. No significant differences were found in plasma levels of saccharin and in urinary excretion of saccharin and sucralose. Consistent with similar intake of dietary inositol from AA-MF and GMP-MF, no significant differences were found in plasma and urine concentrations of myo- and chiro-inositol.

### 3.7. Adequate Status of Iron, Vitamin B-12, and Zinc Based on Hematological Measures

Average concentrations of hemoglobin, plasma ferritin, serum MMA, and zinc were within normal limits (Supplemental Table 4). Consistent with adequate iron status, hemoglobin and ferritin levels were within normal limits for >93% of participants, and >97% of participants met or exceeded the EAR for dietary iron intake. Despite the fact that 13–20% of participants consuming AA-MF or GMP-MF, respectively, did not meet the EAR for dietary zinc, serum zinc concentrations for 94% of participants were within normal limits for both treatments. Given that serum zinc is correlated with dietary zinc intake and a good biomarker of zinc status [34], these data suggest adequate zinc status. In line with adequate vitamin B-12 status, MMA concentrations were within normal limits for >94% of participants, and >90% of participants met or exceeded the EAR for dietary vitamin B-12 intake.

## 4. Discussion

### 4.1. Summary of Intake

Individuals with PKU are at risk for nutrient deficiencies and toxicities due to the restrictive low-Phe diet in combination with medical foods that are supplemented with chemically derived nutrients in variable amounts [35, 36]. We conducted an intervention trial that investigated nutrient status using dietary intakes and biochemical measures in adults and adolescents with classical and variant PKU who showed similar compliance when consuming AA-MF and Glytactin GMP-MF. A list of the main conclusions from this study is detailed in Table 6. For the majority of micronutrients, we found similar total intakes when participants consumed AA-MF or Glytactin GMP-MF and no differences in intakes of micronutrients from natural foods. This indicates that differences in micronutrient intake were driven by the diverse micronutrient supplementation profiles of the medical foods used by our participants. Although participants obtained adequate intakes of most micronutrients based on the EAR, inadequate intakes of potassium for 93% of participants and choline for >40% of participants were observed. In contrast to inadequate intake, participants had excessive intakes (>UL) of chemically derived folic acid and magnesium from medical foods, and >63% of participants had excessive intakes of sodium driven by natural (likely processed) food intake. Nonetheless, vitamin and mineral supplementation of medical foods is necessary in order to prevent nutrient deficiency in PKU. Our data showed that at least 70% of participants would obtain inadequate intakes for 11 vitamins and minerals without supplemented medical foods (biotin, choline, pantothenate, vitamins D and E, potassium, calcium, iodine, magnesium, selenium, and zinc).

### 4.2. Common Nutrients of Concern in PKU

Adequate nutrient status in PKU relies on compliance with medical food supplemented with vitamins and minerals due to the low natural protein intake tolerated with the low-Phe diet [4]. Micronutrient deficiency, as evidenced by dietary intake and biochemical measures, has been reported for iron, vitamin B-12, and zinc [35, 36] but can generally be avoided with compliance with medical foods that are supplemented with these micronutrients [35–38]. Consistent with adequate iron and vitamin B-12 status, Pinto et al. found similar levels of ferritin, transferrin, hemoglobin, and vitamin B-12 that were within normal limits in 11 subjects with PKU consuming AA-MF in combination with Glytactin GMP-MF for ∼13 months [21]. Our data also suggest that participants demonstrated adequate status of iron, vitamin B-12, and zinc as evidenced by dietary intakes that meet or exceed the EAR and relevant biomarker concentrations within normal limits for the majority of participants.

Excessive folic acid intake supported by high levels of folate or folic acid biomarkers is noted to be of concern in PKU [18, 36, 39]. Evans et al. reported folate intakes that were 201–267% of the Recommended Nutrient Intake in 51 children, aged 1–16 years, supported by high serum folate concentrations that were above normal limits in 83% of subjects [18]. Crujeiras et al. showed high folate levels in 39% of 156 children and adults with PKU, aged 7 months–42 years. We observed dietary folic acid intakes from AA-MF and Glytactin GMP-MF that exceeded the UL in >27% of participants, but folate or folic acid concentrations were not measured. In contrast, Pinto et al. did not find high folic acid concentrations in subjects consuming AA-MF and Glytactin GMP-MF, but dietary intakes of folate and folic acid were not reported [21]. Andrade et al. found altered methylation capacity resulting in low homocysteine concentrations in 42 subjects with PKU compared to 40 controls, which they attribute to high intakes of folic acid and vitamin B-12 from low-Phe medical food products [40]. Dobrowolski et al. reported methylome repatterning in the brain demonstrated by aberrant DNA methylation in brain tissue of subjects and mice with PKU [41, 42]. Given the role of folate in one-carbon metabolism, it is possible that excessive intake of folic acid from both AA-MF and GMP-MF and excessive intake of sulfur-containing amino acids from AA-MF could contribute to gene dysregulation in the brain secondary to aberrant DNA methylation [41, 42]. In short, excessive folic acid intake from medical foods might contribute to the neuropathology of PKU.

Selenium is one micronutrient where observations of deficiency and toxicity, supported by plasma selenium and glutathione peroxidase activity, have been reported [18, 35, 36]. Conflicting reports of selenium deficiency and toxicity may be related to a combination of reduced bioavailability of chemically derived selenium and high supplementation of some medical foods with selenium. Nonetheless, >80% of participants in this study obtained intakes of selenium that met the EAR, but biomarkers of selenium status were not measured.

### 4.3. Metabolomics

#### 4.3.1. Vitamin A

Most participants (>90%) were able to reach the EAR for vitamin A without supplementation from medical foods, although status of our participants is unclear. Two studies have come to conflicting conclusions regarding vitamin A status in PKU based on reported dietary intake and utilization of different biomarkers for vitamin A [43, 44]. Colomé et al. reported good vitamin A status based on plasma retinol; however, retinol is homeostatically controlled and is only reduced in cases of severe deficiency [12, 43]. Schulpis et al. reported concerns of hypervitaminosis A based on high plasma *β*-carotene concentrations in 46 children with PKU, who consumed relatively large amounts of carotenoid-rich low-Phe fruits and vegetables compared to age-matched controls [44]. Given the high consumption of carotenoid-rich fruits and vegetables in PKU and the potential for hypervitaminosis A with excess provitamin A intake, high plasma concentrations of retinal and *β*-carotene may be particularly relevant to assessment of vitamin A status in PKU [12, 43].

Most AA-MF and GMP-MF are supplemented with preformed vitamin A in the form of retinyl palmitate or retinyl acetate, which may increase the potential for excessive vitamin A intake. Hypervitaminosis A can increase risk for osteoporosis, liver dysfunction, and immune function alterations [12]. Though vitamin A intakes observed in our participants were high (median intakes, 6996–8089 IU/d), 64–72% of total vitamin A intake was obtained from natural foods. Because the majority of our participants' vitamin A intake likely came from provitamin A carotenoids (*β*-carotene, *α*-carotene, and *β*-cryptoxanthin), participants' high vitamin A intake does not exceed the UL, which includes only preformed vitamin A per current Institute of Medicine standards [25]. Though we observed ∼1.7-fold higher plasma retinal concentrations in PKU subjects, independent of diet and genotype, compared to controls, no conclusions related to hypervitaminosis A can be made, given the limited understanding of evaluation of vitamin A status in the vitamin A field. Given the known complications of skeletal fragility and inflammation in PKU, serum retinyl esters, a newly recognized biomarker of hypervitaminosis A, should be measured to further investigate the concerns of hypervitaminosis A [12]. Lastly, supplementation of medical foods with provitamin A, rather than preformed vitamin A, may be prudent.

#### 4.3.2. Choline

Choline is an essential nutrient that is important for function of all cells, such as structural integrity and signaling of cell membranes, and stem cell proliferation and apoptosis, which can impact brain structure and function, particularly during development [31]. Choline can be obtained from high-protein dietary sources, such as meat, eggs, beans, and nuts, but can also be made via de novo synthesis from phosphatidylcholine in the liver [31]. Considering that most sources of choline are in high-protein foods, individuals with PKU must rely on de novo synthesis and supplementation of medical foods with choline.

Choline intakes were below the EAR in >40% of participants, though participants obtained 1.55-fold greater intakes of dietary choline with Glytactin GMP-MF compared to AA-MF. It was not surprising to find no differences in plasma choline concentration, given its homeostatic control. However, choline appears to be a nutrient of concern in participants with classical PKU as evidenced by the lower plasma betaine concentrations compared to controls and lower dimethylglycine concentrations compared to variant PKU. Given the challenge of evaluating choline status with plasma biomarkers (choline, betaine, and dimethylglycine) and the underestimated DRI for dietary choline, it is reasonable to suggest that choline is a nutrient of concern in PKU based on the low dietary choline intakes of our participants [31].

### 4.4. Strengths and Limitations

Strengths of this study include a crossover study design, metabolomic analyses of both plasma and urine samples, and a comprehensive diet analysis that consisted of macronutrients and 25 vitamins and minerals. Furthermore, unlike the nutrition intervention studies with GMP-MF in patients with PKU, an additional strength of this study is that GMP-MF contributed to 95–100% of medical food intake [21, 45]. Limitations of this study include a short dietary treatment with GMP-MF that consisted of 3 weeks and inclusion of 15 different AA-MF to accommodate participant preference.

## 5. Conclusions

This is the first study to utilize metabolomic analysis of plasma and urine samples and employ a comprehensive diet analysis that estimated 25 vitamins and minerals from medical foods and natural foods to investigate the nutrient status of adults and adolescents with classical and variant PKU consuming AA-MF and Glytactin GMP-MF. Our results are pertinent to early treated adults and adolescents with PKU who are adherent to the low-Phe diet in combination with medical foods. We identified several nutrients of concern related to inadequate intakes (potassium and choline) or excessive intakes (sodium, magnesium, folic acid, and possibly vitamin A). Our data demonstrate that without vitamin and mineral supplementation of medical foods, more than 70% of participants would have inadequate intakes for 11 vitamins and minerals (biotin, choline, pantothenate, vitamins D and E, potassium, calcium, iodine, magnesium, selenium, and zinc). Thus, nutrient status in PKU relies on compliance with medical foods supplemented with vitamins and minerals. Regardless, there are ongoing challenges related to nutrient deficiency and toxicity in PKU due to the minimal intake of natural foods that contain protein in the low-Phe diet, the bioavailability of chemically derived micronutrients, and the diverse micronutrient supplementation profiles of medical foods. More research is necessary to determine optimal supplementation needs of chemically derived micronutrients for individuals with PKU across the lifespan.

## Figures and Tables

**Table 1:** Participant characteristics^1^.

	Adult males	Adult females	Minors^2^	*p*	
				Adult and minors	Adults only
Age, y	28 ± 9 (18–44)	30 ± 7 (23–49)	16 ± 1 (15–17)	0.003	0.64
BMI, kg/m^2^	25.2 ± 3.6	26.9 ± 5.3	23.2 ± 2.4	0.29	0.34
BMI percentile	—	—	69.6 ± 23.7 (30–93)	—	—
Plasma Phe, *µ*mol/L	770 ± 217 (367–1086)	658 ± 337 (201–1360)	706 ± 540 (224–1418)	0.63	0.52
Classical PKU, *n*	8	10	2	—	—
Variant PKU, *n*	2	5	3	—	—
Kuvan™ user, *n*	1	2	2	—	—

^1^Data represent participant characteristic data collected at baseline (visit 1) for our clinical trial, *n* = 30 [8]. Values are means ± SD, and values in parenthesis represent the minimum and maximum. Subanalyses were done to compare adult male and female participants; ^2^of the five participants who were minors (<18 y), two were male and three were female; BMI, body mass index; PKU, phenylketonuria.

**Table 2:** Daily macronutrient intake profiles in combination with AA-MF and GMP-MF^1^.

	Median and percentile nutrient intakes						*p*
	AA-MF			GMP-MF			
	10th	Median	90th	10th	Median	90th	
kcal	1471	2076	2711	1523	2148	3152	0.33
kcal/kg	23	29	43	21	30	46	0.33
kcal from MF	162	393	913	340	694	1007	0.002
kcal from NF	969	1597	2247	905	1532	2467	0.53
g protein	61	76	107	56	81	109	0.81
g protein/kg	0.85	1.10	1.50	0.80	1.10	1.70	1.00
g PE from MF	33	59	75	30	53	73	0.98
g PE from MF/kg	0.47	0.78	1.10	0.45	0.76	1.03	0.94
% PE from MF	34%	77%	84%	40%	71%	86%	0.58
g protein from NF	12	20	46	11	22	58	0.88
mg Phe	487	924	1973	544	1014	2592	0.25
mg Phe from MF	0	0	0	50	85	137	0.0001^†^
mg Phe from NF	487	924	1973	419	929	2524	0.97
g carbohydrate	203	294	406	195	347	473	0.10
g carbohydrate from MF	1	36	125	42	91	166	0.0001^†^
g carbohydrate from NF	150	237	363	127	240	369	0.52
g sugar/d	67	122	243	78	129	227	0.69
g sugar from MF	0	36	102	22	53	109	0.10^†^
% energy from MF from sugar	0%	34%	51%	20%	30%	55%	0.18
g sugar from NF	19	89	170	35	69	153	0.94
g fiber	10	17	39	9	18	28	0.93
g fiber from MF	0	0	17	0	2	4	0.67
g fiber from NF	10	15	24	9	16	28	0.64
g fat	37	64	92	35	65	102	0.62
g fat from MF	0	1	20	0	15	26	0.0004
g fat from NF	31	58	82	23	53	93	0.63
g saturated fat	8	15	26	10	21	31	0.046
g saturated fat from MF	0	0	1	0	4	11	0.005
g saturated fat from NF	8	15	25	5	13	28	0.85
g trans fat^2^	0.1	0.4	1.3	0.03	0.4	1.8	0.66
mg cholesterol^2^	9	30	118	4	40	136	0.76

^1^Nutrient intakes were based on 3-day food records (*n* = 30). Statistical analysis included ANOVA with effects for treatment, genotype, and treatment-genotype interaction. The *p* values in this table represent the treatment comparison; ^2^AA-MF and GMP-MF did not contain trans fat nor cholesterol; ^†^Kruskal–Wallis test was used when data were skewed; AA-MF, amino acid medical food; GMP-MF, glycomacropeptide medical food; MF, medical food; NF, natural food; PE, protein equivalent.

**Table 3:** Modified low-protein daily food intake profiles in combination with AA-MF and GMP-MF^1^.

	Median and percentile nutrient intakes						*p*
	AA-MF			GMP-MF			
	10th	Median	90th	10th	Median	90th	
Energy, kcal/d	73	211	463	85	257	690	0.31
Protein, g/d	0.1	0.6	1.3	0.2	0.4	1.6	0.71
Phe, mg/d	3	13	60	3	20	93	0.32
Carbohydrate, g/d	17	51	103	14	62	125	0.49^‡^
Fiber, g/d	0	0.3	5	0	1	6	0.13
Fat, g/d	0	1	5	0.5	3	18	0.13^‡^
Saturated fat, g/d	0	0.2	2	0	0.5	4	0.25^‡^

^1^Nutrient intakes from MLPF were based on 3-day food records (*n* = 13; classical PKU, *n* = 10; variant PKU, *n* = 3). Participants that did not consume MLPF at the end of both treatments were removed from the analysis. MLPF were defined as foods modified to be low in protein and specifically made for individuals with disorders that have dietary protein restrictions. Statistical analysis included ANOVA with effects for treatment, genotype, and treatment-genotype interaction. The *p* values in this table represent the treatment comparison. There were no significant differences due to genotype or the treatment-genotype interaction (data not shown); ^‡^Kruskal–Wallis test was used when data were skewed; MLPF, modified low-protein foods.

**Table 4:** Treatment comparison of vitamin and additive nutrient intake distributions in comparison with reference intake cutoffs with AA-MF and GMP-MF^1^.

Vitamins	AA-MF						GMP-MF					*p*
	*n*	Percentile nutrient intakes			Inadequate and excessive intakes^2^		Percentile nutrient intakes			Inadequate and excessive intakes		
		10th	Median	90th	<EAR or AI	>UL	10th	Median	90th	<EAR or AI	>UL	
*Fat-soluble vitamins*												
Vitamin A, IU	30	3454	6996	17,572	0%	—	2479	8089	16,320	3%	—	0.39
Vitamin A from MF	30	0	2642	4037	—	0%	0	2246	4008	—	0%	0.15
Vitamin A from MF^3^	21	1543	2840	4063	—	—	1348	2295	4043	—	—	0.60
Vitamin A from NF^4^	30	1656	5061	14,604	7%	—	983	5232	13,795	10%	—	0.99
Vitamin D, IU	30	148	562	1941	33%	3%	36	511	1243	33%	3%	0.58^†^
Vitamin D from MF	30	0	396	960	—	—	0	485	868	—	—	0.99^†^
Vitamin D from MF	24	234	599	960	—	—	0	500	929	—	—	0.50^†^
Vitamin D from NF	30	2	27	644	90%	—	3	30	159	93%	—	1.00
Vitamin E, IU	30	13	29	55	13%	—	7	25	37	23%	—	0.002
Vitamin E from MF	30	0	18	28	—	0%	0	15	23	—	0%	0.14^†^
Vitamin E from MF	21	13	18	27	—	—	8	15	25	—	—	0.01
Vitamin E from NF	30	6	10	38	80%	—	4	10	20	87%	—	0.16^†^
Vitamin K, µg	30	119	160	412	3%	ND^5^	59	190	393	23%	ND	0.27
Vitamin K from MF	30	0	75	112	—	—	0	74	131	—	—	0.91^†^
Vitamin K from MF	21	55	75	109	—	—	42	75	134	—	—	0.75
Vitamin K from NF	30	29	103	311	47%	—	20	102	308	53%	—	0.39

*Water-soluble vitamins*												
Vitamin C, mg	30	78	161	276	3%	0%	45	164	296	13%	0%	0.86
Vitamin C from MF	30	0	61	130	—	—	0	74	129	—	—	0.46^†^
Vitamin C from MF	21	42	62	133	—	—	44	81	131	—	—	0.19
Vitamin C from NF	30	19	94	212	37%	—	27	72	197	43%	—	0.62
Thiamin, mg	30	1.2	2.5	6.5	3%	ND	0.9	2.0	2.7	13%	ND	0.03^†^
Thiamin from MF	30	0	1.3	3.9	—	—	0	1.0	1.7	—	—	0.01
Thiamin from MF	21	0.6	1.3	3.7	—	—	0.6	1.0	1.8	—	—	0.02
Thiamin from NF	30	0.5	1.0	3.0	33%	—	0.4	0.9	1.7	63%	—	0.15^†^
Riboflavin, mg	30	1.0	2.6	4.0	10%	ND	1.8	2.9	4.7	0%	ND	0.33^†^
Riboflavin from MF	30	0	1.5	2.8	—	—	1.1	1.8	2.9	—	—	0.12
Riboflavin from MF	25	0.7	1.5	2.9	—	—	1.0	1.7	2.8	—	—	0.41
Riboflavin from NF	30	0.5	1.0	2.1	47%	—	0.4	0.8	1.9	63%	—	0.31^†^
Niacin, mg	30	16	31	65	3%	—	16	32	42	3%	—	0.87^†^
Niacin from MF	30	0	16	55	—	20%	7	18	26	—	0%	0.52^†^
Niacin from MF	25	5	21	57	—	—	7	18	27	—	—	0.68^†^
Niacin from NF	30	7	13	26	37%	—	6	12	25	43%	—	0.19
Vitamin B-6, mg	30	1.9	3.1	5.7	3%	0%	1.8	3.1	5.7	0%	0%	0.65^†^
Vitamin B-6 from MF	30	0	1.7	2.8	—	—	1.1	1.8	2.9	—	—	0.28
Vitamin B-6 from MF	25	1	1.7	2.9	—	—	1.1	1.8	2.9	—	—	0.62
Vitamin B-6 from NF	30	0.7	1.5	2.8	27%	—	0.5	1.2	4.3	37%	—	0.34^†^
Folate DFE, µg	30	504	1159	1902	3%	—	593	1193	1461	3%	—	0.58^†^
Folate DFE from MF	30	0	743	1507	—	37%	192	733	1126	—	27%	0.80^†^
Folate DFE from MF	24	382	851	1512	—	—	457	800	1141	—	—	0.06
Folate DFE from NF	30	160	347	847	40%	—	116	364	790	43%	—	0.35
Vitamin B-12, mg	30	2.3	6.5	11.8	10%	ND	4.1	7.1	13.9	0%	ND	0.23^†^
Vitamin B-12 from MF	30	0	5.1	6.8	—	—	2.4	4.8	9.4	—	—	0.14
Vitamin B-12 from MF	25	2.2	5.4	7.0	—	—	2.4	4.5	10.8	—	—	0.71
Vitamin B-12 from NF	30	0.1	1.5	8.1	53%	—	0.2	1.1	8.7	63%	—	0.71^†^
Choline, mg	30	74	311	674	63%	0%	71	512	868	40%	0%	0.02
Choline from MF	30	0	277	628	—	—	0	430	780	—	—	0.03
Choline from MF	21	111	414	588	—	—	253	501	806	—	—	0.003
Choline from NF	30	25	65	109	100%	—	36	63	172	100%	—	0.15
Pantothenate, mg	30	4	9	15	17%	ND	6	10	16	7%	ND	0.47^†^
Pantothenate from MF	30	0	5	13	—	—	4	7	11	—	—	0.21
Pantothenate from MF	25	3	5	13	—	—	4	7	12	—	—	0.96
Pantothenate from NF	30	1	3	7	83%	—	1	2	4	93%	—	0.38^†^
Biotin, mg	30	18	67	242	23%	ND	27	104	428	13%	ND	0.14
Biotin from MF	30	0	42	160	—	—	23	67	359	—	—	0.06
Biotin from MF	25	20	97	160	—	—	22	54	403	—	—	0.69
Biotin from NF	30	2	6	126	83%	—	1	4	13	93%	—	0.32^†^

*Other*												
Carnitine from MF, g	30	0.030	0.057	0.100	—	—	<0.001	<0.001	<0.001	—	—	<0.0001^†^
Taurine from MF, g	30	0.065	0.130	0.300	—	—	0.085	0.160	0.258	—	—	0.27
Inositol from MF, g	30	0	0.080	0.135	—	—	0	0.005	0.048	—	—	<0.0001^†^

^1^Nutrient intakes were based on 3-day food records (*n* = 30); statistical analysis included ANOVA with effects for treatment, genotype (classical or variant PKU), and treatment-genotype interaction. The *p* values in this table represent the treatment comparison; ^2^inadequate and excessive intakes are expressed as a percentage of 30 subjects. United States' Dietary Reference Intake (DRI) cutoffs, based on the AMDR, EAR, AI, and UL, were compared to nutrient intakes per the sex and age of each individual subject [25, 26]. The United States Dietary Guidelines for Americans 2015–2020 reference cutoffs were used to evaluate dietary saturated fat and sugar intake of subjects [24]; ^3^subanalyses for micronutrient intakes from medical foods were conducted due to the high rates of use of AA-MF that lacked micronutrient supplementation. These subanalyses aimed to compare micronutrient intakes from medical foods that were supplemented with micronutrients. Due to the diverse micronutrient supplementation profiles of medical foods, sample sizes for each micronutrient vary; ^4^natural foods were defined as all foods and beverages that were not medical foods intended for the treatment of PKU; ^5^per the DRI, a UL for select nutrients has not been determined due to a lack of scientific evidence; ^†^Kruskal–Wallis test was used when data were skewed; AMDR, acceptable macronutrient distribution range; AI, adequate intake; AA-MF, amino acid medical food; DFE, dietary folate equivalents; DGA, Dietary Guidelines for Americans; EAR, estimated average requirement; GMP-MF, glycomacropeptide medical food; MF, medical food; ND, not determined; NF, natural food; PKU, phenylketonuria; UL, upper tolerable intake levels.

**Table 5:** Treatment comparison of mineral intake distributions in comparison with reference intake cutoffs with AA-MF and GMP-MF^1^.

Minerals	AA-MF						GMP-MF					*p*
	*n*	Percentile nutrient intakes			Inadequate and excessive intakes^2^		Percentile nutrient intakes			Inadequate and excessive intakes		
		10th	Median	90th	<EAR or AI	>UL	10th	Median	90th	<EAR or AI	>UL	
Calcium, mg	30	839	1468	2402	10%	0%	586	1529	2453	20%	7%	1.00^†^
Calcium from MF	30	106	1103	2077	—	—	63	1153	2225	—	—	0.99
Calcium from MF^3^	27	552	1151	2129	—	—	60	1255	2391	—	—	0.62^†^
Calcium from NF^4^	30	183	313	784	93%	—	156	401	726	90%	—	0.80^†^
Copper, µg	30	1.2	1.9	4.2	3%	7%	0.6	1.5	2.7	13%	0%	0.0006^†^
Copper from MF	30	0	1.2	3.0	—	—	0	0.6	1.1	—	—	0.005
Copper from MF	21	0.6	1.2	2.8	—	—	0.4	0.7	1.1	—	—	0.0001
Copper from NF	30	0.5	0.9	3.4	33%	—	0.4	0.8	1.9	40%	—	0.27^†^
Iodine, µg	30	13	140	235	23%	0%	12	153	262	33%	0%	0.66^†^
Iodine from MF	30	0	132	223	—	—	0	141	239	—	—	0.48^†^
Iodine from MF	21	77	137	223	—	—	78	149	241	—	—	0.32
Iodine from NF	30	2	9	42	93%	—	0	8	48	97%	—	0.26
Iron, mg	30	14	25	43	0%	3%	9	23	31	3%	0%	0.02
Iron from MF	30	0	15	31	—	—	0	14	23	—	—	0.14^†^
Iron from MF	21	10	16	31	—	—	8	14	23	—	—	0.02
Iron from NF	30	5	8	22	37%	—	4	8	17	33%	—	0.15
Magnesium, mg	30	281	501	746	13%	—	196	514	782	23%	—	0.78^†^
Magnesium from MF	30	32	320	570	—	37%	41	353	578	—	53%	0.77^†^
Magnesium from MF	27	126	321	570	—	—	38	354	600	—	—	0.80^†^
Magnesium from NF	30	110	171	320	90%	—	77	165	292	87%	—	0.41
Manganese, mg	30	1.9	3.9	7.1	10%	0%	1.8	3.8	5.3	13%	3%	0.26
Manganese from MF	30	0	1.6	3.4	—	—	0	1.6	3.0	—	—	0.84^†^
Manganese from MF	21	0.9	1.7	3.4	—	—	0.6	1.9	3.1	—	—	0.41^†^
Manganese from NF	30	1.1	2.2	6.3	40%	—	0.9	1.9	3.7	50%	—	0.10
Phosphorus, mg	30	952	1616	2431	7%	0%	774	1536	2204	10%	0%	0.60
Phosphorus from MF	30	81	972	2036	—	—	253	965	1708	—	—	0.71^†^
Phosphorus from MF	27	531	1032	2047	—	—	240	984	1800	—	—	0.26
Phosphorus from NF	30	329	526	1007	73%	—	259	538	1064	67%	—	0.80
Potassium, mg	30	1809	2810	4179	93%	ND^5^	2014	3350	4540	93%	ND	0.15
Potassium from MF	30	0	600	2558	—	—	718	1146	1781	—	—	0.08^†^
Potassium from MF	25	60	880	2673	—	—	680	1120	1815	—	—	0.46^†^
Potassium from NF	30	938	1910	3011	97%	—	1077	1867	3204	100%	—	1.00^†^
Selenium, µg	30	54	93	133	7%	0%	25	88	142	17%	0%	0.50^†^
Selenium from MF	30	9	58	89	—	—	0	55	89	—	—	0.34^†^
Selenium from MF	23	26	59	89	—	—	29	60	93	—	—	0.31
Selenium from NF	30	12	29	88	83%	—	8	27	103	73%	—	0.95
Sodium, mg	30	1816	2637	3879	7%	63%	2052	3261	4645	7%	83%	0.048
Sodium from MF	30	0	413	1175	—	—	580	1140	2040	—	—	<0.0001^†^
Sodium from MF	25	17	583	1198	—	—	480	1123	2280	—	—	0.0003
Sodium from NF	30	1363	2206	3138	17%	—	1007	2041	3383	23%	—	0.69
Zinc, mg	30	5	16	41	13%	13%	5	13	31	20%	10%	0.12
Zinc from MF	30	0	12	30	—	—	0	9	17	—	—	0.05
Zinc from MF	21	6	12	28	—	—	6	10	17	—	—	0.14^†^
Zinc from NF	30	3	4	17	77%	—	2	4	19	83%	—	0.64^†^

^1^Nutrient intakes were based on 3-day food records (*n* = 30); statistical analysis included ANOVA with effects for treatment, genotype (classical or variant PKU), and treatment-genotype interaction. The *p* values in this table represent the treatment comparison; ^2^United States' Dietary Reference Intake (DRI) cutoffs, based on the AMDR, EAR, AI, and UL, were compared to nutrient intakes per the sex and age of each individual subject [25, 26]; ^3^sub-analyses for micronutrient intakes from medical foods were conducted due to the high rates of use of AA-MF that lacked micronutrient mineral supplementation. These subanalyses aimed to compare micronutrient intakes from medical foods that were supplemented with micronutrients. Due to the diverse micronutrient supplementation profiles of medical foods, sample sizes for each micronutrient vary; ^4^natural foods were defined as all foods and beverages that were not medical foods intended for the treatment of PKU; ^5^per the DRI, a UL for select nutrients has not been determined due to a lack of scientific evidence; ^†^Kruskal–Wallis test was used when data were skewed; AMDR, acceptable macronutrient distribution range; AI, adequate intake; AA-MF, amino acid medical food; DFE, dietary folate equivalents; DGA, Dietary Guidelines for Americans; EAR, estimated average requirement; GMP-MF, glycomacropeptide medical food; MF, medical food; ND, not determined; NF, natural food; PKU, phenylketonuria; UL, upper tolerable intake levels.

**Table 6:** Main study conclusions.

(1)	Similar total dietary intakes of most micronutrients when participants consumed AA-MF or Glytactin GMP-MF and no differences in intakes of micronutrients from natural foods were observed. Thus, differences in micronutrient intakes were driven by the diverse micronutrient supplementation profiles of the medical foods.

(2)	Participants obtained adequate intakes (≥EAR) of most micronutrients. However, inadequate intakes (i.e., <EAR) of potassium for 93% of participants and choline for >40% of participants were observed.

(3)	Participants had excessive intakes (>UL) of chemically derived folic acid and magnesium from medical foods, and >63% of participants had excessive intakes of sodium driven by natural (likely processed) food intake. Average sugar intake as a percentage of energy was 27% and was excessive (>DGA) for 97% of participants.

(4)	Without micronutrient supplementation of medical foods, >70% of participants would have inadequate intakes (≤EAR) for 11 micronutrients (biotin, choline, pantothenate, vitamins D and E, potassium, calcium, iodine, magnesium, selenium, and zinc). Greater than 90% of participants would obtain adequate intake (≥EAR) of vitamin A from natural foods alone due to high intakes of provitamin A carotenoids from green leafy vegetables, squashes, carrots, and tomatoes.

(5)	Of 30 participants, only 13 consumed MLPF with both AA-MF and GMP-MF treatments. MPLF comprised approximately 10% of median calories.

(6)	Increased urinary excretion of sulfate, taurine, and taurine-related metabolites with AA-MF may be related to increased need to excrete sulfur with higher dietary intake of sulfur-containing amino acids, Met and Cys, from AA-MF compared with Glytactin GMP-MF.

## Abbreviations

AA: Amino acid
AA-MF: Amino acid medical foods
AI: Adequate intake
AMDR: Acceptable macronutrient distribution ranges
BMD: Bone mineral density
BMI: Body mass index
DGA: Dietary Guidelines for Americans
DHA: Docosahexaenoic acid
DRI: Dietary Reference Intake
EAR: Estimated average requirements
EPA: Eicosapentaenoic acid
GMP-MF: Glycomacropeptide medical foods
MF: Medical foods
MLPF: Modified low-protein food
MMA: Methylmalonic acid
PAH: Phenylalanine hydroxylase
PE: Protein equivalent
RD: Registered dietitian
UL: Tolerable upper intake level.

## Supplementary Materials

Supplementary Materials Supplemental Table 1: Plasma metabolomics in subjects with PKU consuming AA-MF or GMP-MF compared to controls. Supplemental Table 2: Plasma metabolomics in subjects with classical versus variant PKU consuming AA-MF and GMP-MF compared to controls. Supplemental Table 3: Urine metabolomics in subjects with classical and variant PKU consuming AA-MF and GMP-MF. Supplemental Table 4: Laboratory Measures.

## References

[1] Flydal M. I., Martinez A. (2013). Phenylalanine hydroxylase: function, structure, and regulation. *IUBMB Life*.

[2] Vockley J., Andersson H. C., Antshel K. M. (2014). Phenylalanine hydroxylase deficiency: diagnosis and management guideline. *Genetics in Medicine*.

[3] Bilder D. A., Kobori J. A., Cohen-Pfeffer J. L., Johnson E. M., Jurecki E. R., Grant M. L. (2017). Neuropsychiatric comorbidities in adults with phenylketonuria: a retrospective cohort study. *Molecular Genetics and Metabolism*.

[4] Singh R. H., Rohr F., Frazier D. (2014). Recommendations for the nutrition management of phenylalanine hydroxylase deficiency. *Genetics in Medicine*.

[5] Etzel M. R. (2004). Manufacture and use of dairy protein fractions. *Journal of Nutrition*.

[6] Laclair C. E., Ney D. M., MacLeod E. L., Etzel M. R. (2009). Purification and use of glycomacropeptide for nutritional management of phenylketonuria. *Journal of Food Science*.

[7] van Calcar S. C., MacLeod E. L., Gleason S. T. (2009). Improved nutritional management of phenylketonuria by using a diet containing glycomacropeptide compared with amino acids. *American Journal of Clinical Nutrition*.

[8] Ney D. M., Stroup B. M., Clayton M. K. (2016). Glycomacropeptide for nutritional management of phenylketonuria: a randomized, controlled, crossover trial. *American Journal of Clinical Nutrition*.

[9] Singh R. H., Cunningham A. C., Mofidi S. (2016). Updated, web-based nutrition management guideline for PKU: an evidence and consensus based approach. *Molecular Genetics and Metabolism*.

[10] Lammardo A. M., Robert M., Rocha J. C. (2013). Main issues in micronutrient supplementation in phenylketonuria. *Molecular Genetics and Metabolism*.

[11] Jans J. J., de Sain-van der Velden M. G. M., van Hasselt P. M. (2013). Supplementation with a powdered blend of PUFAs normalizes DHA and AA levels in patients with PKU. *Molecular Genetics and Metabolism*.

[12] Tanumihardjo S. A., Russell R. M., Stephensen C. B. (2016). Biomarkers of nutrition for development (BOND)-vitamin A review. *Journal of Nutrition*.

[13] Borel P. (2003). Factors affecting intestinal absorption of highly lipophilic food microconstituents (fat-soluble vitamins, carotenoids and phytosterols). *Clinical Chemistry and Laboratory Medicine*.

[14] Jakeman S. A., Henry C. N., Martin B. R. (2016). Soluble corn fiber increases bone calcium retention in postmenopausal women in a dose-dependent manner: a randomized crossover trial. *American Journal of Clinical Nutrition*.

[15] Whisner C. M., Martin B. R., Nakatsu C. H. (2016). Soluble corn fiber increases calcium absorption associated with shifts in the gut microbiome: a randomized dose-response trial in free-living pubertal females. *Journal of Nutrition*.

[16] Fairweather-Tait S. J., Collings R., Hurst R. (2010). Selenium bioavailability: current knowledge and future research requirements. *American Journal of Clinical Nutrition*.

[17] Öhrvik V. E., Büttner B. E., Rychlik M., Lundin E., Witthöft C. M. (2010). Folate bioavailability from breads and a meal assessed with a human stable-isotope area under the curve and ileostomy model. *American Journal of Clinical Nutrition*.

[18] Evans S., Daly A., MacDonald J. (2014). The micronutrient status of patients with phenylketonuria on dietary treatment: an ongoing challenge. *Annals of Nutrition and Metabolism*.

[19] Demirdas S., van Spronsen F. J., Hollak C. E. M. (2017). Micronutrients, essential fatty acids and bone health in phenylketonuria. *Annals of Nutrition and Metabolism*.

[20] Vugteveen I., Hoeksma M., Bjorke Monsen A. L. (2011). Serum vitamin B_12_ concentrations within reference values do not exclude functional vitamin B_12_ deficiency in PKU patients of various ages. *Molecular Genetics and Metabolism*.

[21] Pinto A., Almeida M. F., Ramos P. C. (2017). Nutritional status in patients with phenylketonuria using glycomacropeptide as their major protein source. *European Journal of Clinical Nutrition*.

[22] Wilson K., Charmchi P., Dworetzsky B. (2016). *State Statutes and Regulations on Dietary Treatments of Disorders Identified Through Newborn Screening*.

[23] Institute of Medicine (2005). *Dietary Reference Intake for Energy, Carbohydrate, Fiber, Fat, Fatty Acids, Cholesterol, Protein and Amino Acids (Macronutrients)*.

[24] U.S. Department of Health and Human Services and U.S. Department of Agriculture (2015). *Dietary Guidelines for Americans 2015–2020*.

[25] Institute of Medicine (2006). *Dietary Reference Intakes: The Essential Guide to Nutrient Requirements*.

[26] Institute of Medicine (2011). *Dietary Reference Intakes for Calcium and Vitamin D*.

[27] Ney D. M., Murali S. G., Stroup B. M. (2017). Metabolomic changes demonstrate reduced bioavailability of tyrosine and altered metabolism of tryptophan via the kynurenine pathway with ingestion of medical foods in phenylketonuria. *Molecular Genetics and Metabolism*.

[28] Stroup B. M. (2017). *Nutritional management of phenylketonuria with glycomacropeptide medical foods*.

[29] Stroup B. M., Murali S. G., Nair N. (2017). Dietary amino acid intakes associated with a low-phenylalanine diet combined with amino acid medical foods and glycomacropeptide medical foods and neuropsychological outcomes in subjects with phenylketonuria. *Data in Brief*.

[30] Stroup B. M., Sawin E. A., Murali S. G., Binkley N., Hansen K. E., Ney D. M. (2017). Amino acid medical foods provide a high dietary acid load and increase urinary excretion of renal net acid, calcium, and magnesium compared with glycomacropeptide medical foods in phenylketonuria. *Journal of Nutrition and Metabolism*.

[31] Zeisel S. H. (2006). Choline: critical role during fetal development and dietary requirements in adults. *Annual Review of Nutrition*.

[32] WHO (2007). Protein and amino acid requirements in human nutrition.

[33] Dominy J. E., Hwang J., Guo S., Hirschberger L. L., Zhang S., Stipanuk M. H. (2008). Synthesis of amino acid cofactor in cysteine dioxygenase is regulated by substrate and represents a novel post-translational regulation of activity. *Journal of Biological Chemistry*.

[34] Lowe N. M., Fekete K., Decsi T. (2009). Methods of assessment of zinc status in humans: a systematic review. *American Journal of Clinical Nutrition*.

[35] Robert M., Rocha J. C., van Rijn M. (2013). Micronutrient status in phenylketonuria. *Molecular Genetics and Metabolism*.

[36] MacDonald A., Rocha J. C., van Rijn M., Feillet F. (2011). Nutrition in phenylketonuria. *Molecular Genetics and Metabolism*.

[37] Fisberg R. M., da Silva-Femandes M. E., Fisberg M., José Schmidt B. (1999). Plasma zinc, copper, and erythrocyte superoxide dismutase in children with phenylketonuria. *Nutrition*.

[38] Dobbelaere D., Michaud L., Debrabander A. (2003). Evaluation of nutritional status and pathophysiology of growth retardation in patients with phenylketonuria. *Journal of Inherited Metabolic Disease*.

[39] Crujeiras V., Aldamiz-Echevarria L., Dalmau J. (2015). Vitamin and mineral status in patients with hyperphenylalaninemia. *Molecular Genetics and Metabolism*.

[40] Andrade F., López-Suárez O., Llarena M., Couce M. L., Aldámiz-Echevarría L. (2017). Influence of phenylketonuria’s diet on dimethylated arginines and methylation cycle. *Medicine*.

[41] Dobrowolski S. F., Lyons-Weiler J., Spridik K. (2015). Altered DNA methylation in PAH deficient phenylketonuria. *Molecular Genetics and Metabolism*.

[42] Dobrowolski S. F., Lyons-Weiler J., Spridik K., Vockley J., Skvorak K., Biery A. (2016). DNA methylation in the pathophysiology of hyperphenylalaninemia in the PAH^enu2^ mouse model of phenylketonuria. *Molecular Genetics and Metabolism*.

[43] Colomé C., Artuch R., Vilaseca M. A. (2003). Lipophilic antioxidants in patients with phenylketonuria. *American Journal of Clinical Nutrition*.

[44] Schulpis K. H., Tsakiris S., Karikas G. A., Moukas M., Behrakis P. (2003). Effect of diet on plasma total antioxidant status in phenylketonuric patients. *European Journal of Clinical Nutrition*.

[45] Daly A., Evans S., Chahal S., Santra S., MacDonald A. (2017). Glycomacropeptide in children with phenylketonuria: does its phenylalanine content affect blood phenylalanine control?. *Journal of Human Nutrition and Dietetics*.

