# Supplementary material for: Metabolomic Insights into the Nutritional Status of Adults and Adolescents with Phenylketonuria Consuming a Low-Phenylalanine Diet in Combination with Amino Acid and Glycomacropeptide Medical Foods
Source: J Nutr Metab. 2017 Dec 31;2017:6859820. doi: 10.1155/2017/6859820 (PMC5804357; doi:10.1155/2017/6859820)
Supplement: Supplementary Materials — Supplemental Table 1: Plasma metabolomics in subjects with PKU consuming AA-MF or GMP-MF compared to controls. Supplemental Table 2: Plasma metabolomics in subjects with classical versus variant PKU consuming AA-MF and GMP-MF compared to controls. Supplemental Table 3: Urine metabolomics in subjects with classical and variant PKU consuming AA-MF and GMP-MF. Supplemental Table 4: Laboratory Measures. [file 6859820.f1.docx]

SUPPLEMENTAL TABLE 1. Plasma metabolomics in subjects with PKU consuming AA-MF or GMP-MF compared to controls.

|  | | **GMP-MF**  **AA-MF** | | **GMP-MF**  **Control** | | **AA-MF**  **Control** | | **Mean Values** | | |
| --- | --- | --- | --- | --- | --- | --- | --- | --- | --- | --- |
| **Sub-Pathway** | **Biochemical Name** | **Fold of Change** | ***P*** | **Fold of Change** | ***P*** | **Fold of Change** | ***P*** | **GMP-MF** | **AA-MF** | **Control** |
| Met, Cys, SAM and Taurine Metabolism | betaine | 1.17 | 0.0668 | 0.98 | 0.7409 | 0.85 | 0.1002 | 1.0239 | 0.8826 | 1.0399 |
|  | dimethylglycine | 1.17 | 0.1965 | 1.12 | 0.5769 | 0.95 | 0.7228 | 1.1536 | 0.9740 | 1.0288 |
|  | choline | 1.06 | 0.5789 | 1.06 | 0.4464 | 1.02 | 0.9102 | 1.0846 | 1.0447 | 1.0271 |
|  | taurine | 0.93 | 0.2896 | 0.91 | 0.3384 | 1.05 | 0.9401 | 0.9294 | 1.0745 | 1.0228 |
| Nicotinamide Metabolism | nicotinamide | 1.13 | 0.8303 | 1.47 | 0.0566 | 1.42 | 0.0760 | 1.4392 | 1.3845 | 0.9758 |
| Pantothenate and CoA Metabolism | pantothenate | 1.15 | 0.6333 | **1.48** | **0.0105** | **1.44** | **0.0576** | 1.2396 | 1.2082 | 0.8399 |
| Tocopherol Metabolism | alpha-tocopherol | 0.99 | 0.6994 | 0.88 | 0.1562 | 0.92 | 0.3890 | 0.9100 | 0.9528 | 1.0391 |
|  | delta-tocopherol | 1.26 | 0.4404 | 1.52 | 0.3645 | 1.08 | 0.2569 | 1.2269 | 0.8732 | 0.8069 |
|  | gamma-CEHC | 1.42 | 0.6184 | **1.98** | **0.0315** | 1.68 | 0.0716 | 1.5063 | 1.2803 | 0.7623 |
|  | gamma-CEHC glucuronide | 1.15 | 0.7180 | **2.93** | **0.0019** | **3.19** | **0.0009** | 1.2408 | 1.3507 | 0.4232 |
|  | alpha-CEHC sulfate | **0.60** | **0.0085** | **1.23** | **0.0691** | **2.56** | **0.0012** | 0.7343 | 1.5254 | 0.5966 |
| Vitamin A Metabolism | retinol (Vit. A) | 1.01 | 1.0000 | 1.16 | 0.1728 | 1.17 | 0.1764 | 1.0187 | 1.0198 | 0.8747 |
|  | retinal | 1.09 | 0.5547 | **1.72** | **0.0006** | **1.69** | **0.0053** | 1.3974 | 1.3720 | 0.8120 |
|  | β-cryptoxanthin | 1.01 | 0.8664 | **0.29** | **0.0045** | **0.31** | **0.0064** | 0.8409 | 0.8812 | 2.8722 |
|  | carotene diol (1)^1^ | 1.00 | 0.7091 | **0.52** | **0.0511** | 0.57 | 0.0921 | 0.8275 | 0.9091 | 1.5858 |
|  | carotene diol (2)^1^ | 1.07 | 0.4557 | 0.62 | 0.1507 | 0.63 | 0.1442 | 0.9189 | 0.9271 | 1.4792 |
|  | carotene diol (3)^1^ | 0.92 | 0.2052 | **0.37** | **0.0228** | **0.41** | **0.0540** | 0.8305 | 0.9299 | 2.2699 |
| Vitamin B-6 Metabolism | pyridoxal | 0.99 | 0.7235 | 1.08 | 0.1851 | 1.11 | 0.1564 | 1.0789 | 1.1050 | 0.9982 |
|  | pyridoxate | 0.97 | 0.5065 | 1.06 | 0.2563 | 1.13 | 0.1659 | 1.0396 | 1.1114 | 0.9806 |
| Inositol Metabolism | myo-inositol | 0.88 | 0.1698 | 0.81 | 0.1561 | 1.02 | 0.7577 | 0.9096 | 1.1512 | 1.1286 |
|  | chiro-inositol | 1.98 | 0.9314 | 0.57 | 0.3002 | 1.05 | 0.4336 | 0.5104 | 0.9488 | 0.8994 |
| Food Component/Plant | acesulfame | 15.34 | 0.9867 | 2.89 | 0.0678 | 3.00 | 0.0679 | 0.6842 | 0.7116 | 0.2371 |
|  | erythritol | **34.82** | **0.0190** | **44.99** | **0.0060** | 2.97 | 0.1091 | 36.4712 | 2.4110 | 0.8107 |
|  | saccharin | 3.04 | 0.0911 | 1.00 | 0.3783 | 0.55 | 0.7950 | 1.0636 | 0.5825 | 1.0589 |

Mean values are scaled intensity in participants with PKU consuming AA-MF or GMP-MF (n=10) compared to controls (n=15).

^1^Alpha-CEHC, also known as (2,5,7,8-tetramethyl-2-(2'-carboxyethyl)-6-hydroxychroman), is a major water-soluble α-tocopherol metabolite and can be conjugated to other metabolites, such as alpha-CEHC sulfate. Gamma-CEHC, also known as (3-(2,7,8-Trimethyl-3,4-dihydro-2H-chromen-2-yl) propanoate), is a major water-soluble γ-tocopherol metabolite and can be conjugated to other metabolites, such as gamma-CEHC glucuronide.

^2^Pure standards to precisely identify carotene diols 1-3 were not available from Metabolon; and thus, exact biochemical identification was not determined. Nonetheless, plasma carotene diols 1 and 3 were lower in subjects with PKU consuming GMP-MF and AA-MF compared to controls. AA-MF, amino acid medical foods; GMP-MF, glycomacropeptide medical foods; PKU, phenylketonuria.

SUPPLEMENTAL TABLE 2. Plasma metabolomics in subjects with classical versus variant PKU consuming AA-MF and GMP-MF compared to controls.

|  | | **V**  **C** | | **V**  **Control** | | **C**  **Control** | | **Mean Values** | | |
| --- | --- | --- | --- | --- | --- | --- | --- | --- | --- | --- |
| **Sub-Pathway** | **Biochemical Name** | **Fold of Change** | ***P*** | **Fold of Change** | ***P*** | **Fold of Change** | ***P*** | **V** | **C** | **Control** |
| Met, Cys, SAM and Taurine Metabolism | betaine | 1.20 | 0.2751 | 1.00 | 0.7885 | **0.83** | **0.0422** | 1.0413 | 0.8652 | 1.0399 |
|  | dimethylglycine | **1.46** | **0.0133** | 1.23 | 0.1534 | 0.84 | 0.1539 | 1.2611 | 0.8665 | 1.0288 |
|  | choline | 1.14 | 0.0759 | 1.11 | 0.0796 | 0.97 | 0.5574 | 1.1353 | 0.9941 | 1.0271 |
|  | taurine | 1.18 | 0.2475 | 1.06 | 0.7576 | 0.90 | 0.2862 | 1.0841 | 0.9197 | 1.0228 |
| Nicotinamide Metabolism | nicotinamide | 1.34 | 0.1435 | **1.66** | **0.0113** | 1.24 | 0.2373 | 1.6177 | 1.2061 | 0.9758 |
| Pantothenate and CoA Metabolism | pantothenate | 1.18 | 0.6162 | **1.58** | **0.0322** | **1.34** | **0.0205** | 1.3248 | 1.1230 | 0.8399 |
| Tocopherol Metabolism | alpha-tocopherol | **1.25** | **0.0348** | 1.00 | 0.8171 | **0.80** | 0.0540 | 1.0344 | 0.8284 | 1.0391 |
|  | delta-tocopherol | 0.62 | 0.1948 | 0.99 | 0.3343 | 1.61 | 0.2084 | 0.7999 | 1.3002 | 0.8069 |
|  | gamma-CEHC | 1.17 | 0.2443 | **1.97** | **0.0028** | 1.69 | 0.2034 | 1.5002 | 1.2864 | 0.7623 |
|  | gamma-CEHC glucuronide | 1.53 | 0.1023 | **3.70** | **0.00001** | **2.42** | **0.0138** | 1.5667 | 1.0248 | 0.4232 |
|  | alpha-CEHC sulfate | **1.67** | **0.0277** | **2.37** | **0.0001** | 1.42 | 0.1463 | 1.4120 | 0.8477 | 0.5966 |
| Vitamin A Metabolism | retinol (Vit. A) | 1.17 | 0.1738 | **1.26** | **0.0370** | 1.07 | 0.4988 | 1.0999 | 0.9386 | 0.8747 |
|  | retinal | 0.98 | 0.9931 | **1.69** | **0.0015** | **1.72** | **0.0026** | 1.3730 | 1.3964 | 0.8120 |
|  | β-cryptoxanthin | 0.94 | 0.6652 | **0.29** | **0.0036** | **0.31** | **0.0073** | 0.8342 | 0.8879 | 2.8722 |
|  | carotene diol (1) | 0.94 | 0.9182 | **0.53** | **0.0534** | 0.56 | 0.0859 | 0.8412 | 0.8954 | 1.5858 |
|  | carotene diol (2) | 0.89 | 0.8762 | 0.59 | 0.0984 | 0.66 | 0.2015 | 0.8692 | 0.9769 | 1.4792 |
|  | carotene diol (3) | 1.04 | 0.5983 | **0.40** | **0.0410** | **0.38** | **0.0306** | 0.8987 | 0.8617 | 2.2699 |
| Vitamin B-6 Metabolism | pyridoxal | 1.06 | 0.3546 | 1.13 | 0.0796 | 1.06 | 0.3177 | 1.1261 | 1.0579 | 0.9982 |
|  | pyridoxate | 0.93 | 0.9930 | 1.06 | 0.1653 | 1.14 | 0.2469 | 1.0355 | 1.1156 | 0.9806 |
| Inositol Metabolism | myo-inositol | 1.05 | 0.3779 | 0.93 | 0.6141 | 0.89 | 0.2488 | 1.0532 | 1.0077 | 1.1286 |
|  | chiro-inositol | 2.36 | 0.4886 | 1.14 | 0.6375 | 0.48 | 0.1432 | 1.0254 | 0.4339 | 0.8994 |
| Food Component/Plant | acesulfame | 1.21 | 0.4017 | **3.22** | **0.0226** | **2.66** | **0.1640** | 0.7642 | 0.6317 | 0.2371 |
|  | erythritol | 0.81 | 0.5689 | **21.48** | **0.0175** | 26.48 | 0.0811 | 17.4167 | 21.4656 | 0.8107 |
|  | saccharin | 1.48 | 0.9086 | 0.93 | 0.7093 | 0.63 | 0.7672 | 0.9831 | 0.6630 | 1.0589 |

Mean values are scaled intensity in participants with classical PKU (n=5) or variant PKU (n=5) compared to controls (n=15).

^1^Alpha-CEHC, also known as (2,5,7,8-tetramethyl-2-(2'-carboxyethyl)-6-hydroxychroman), is a major water-soluble α-tocopherol metabolite and can be conjugated to other metabolites, such as alpha-CEHC sulfate. Gamma-CEHC, also known as (3-(2,7,8-Trimethyl-3,4-dihydro-2H-chromen-2-yl) propanoate), is a major water-soluble γ-tocopherol metabolite and can be conjugated to other metabolites, such as gamma-CEHC glucuronide. C, classical phenylketonuria; PKU, phenylketonuria; V, variant phenylketonuria.

SUPPLEMENTAL TABLE 3. Urine metabolomics in subjects with classical and variant PKU consuming AA-MF and GMP-MF.

|  | | **ANOVA Contrasts** | | **Mean Values** | | **ANOVA Contrasts** | | **Mean Values** | |
| --- | --- | --- | --- | --- | --- | --- | --- | --- | --- |
|  |  | **GMP-MF**  **AA-MF** | | **GMP-MF** | **AA-MF** | **V**  **C** | | **V** | **C** |
| **Sub Pathway** | **Biochemical Name** | **Fold of Change** | ***P*** |  |  | **Fold of Change** | ***P*** |  |  |
| Met, Cys, SAM and Taurine Metabolism | betaine | 1.04 | 0.9723 | 1.1891 | 1.1492 | 0.85 | 0.5913 | 1.0820 | 1.2781 |
|  | choline | 1.04 | 0.9999 | 1.074 | 1.082 | 1.31 | 0.1986 | 1.204 | 0.919 |
|  | dimethylglycine | 1.18 | 0.2678 | 1.0508 | 0.9153 | 1.13 | 0.7313 | 1.0376 | 0.9148 |
|  | taurine | **0.32** | **0.0037** | **0.500** | **1.857** | 0.53 | 0.0787 | 0.8427 | 1.5981 |
| Ascorbate and Aldarate Metabolism | ascorbate | 53.81 | 0.4412 | 3.773 | 2.888 | 0.79 | 0.1581 | 2.9807 | 3.7681 |
|  | dehydroascorbate | 5.27 | 0.6126 | 4.993 | 3.882 | 0.67 | 0.1071 | 3.626 | 5.451 |
| Nicotinamide Metabolism | nicotinamide | 1.11 | 0.7847 | 1.058 | 1.064 | **1.57** | **0.0146** | **1.264** | **0.807** |
| Pantothenate and CoA Metabolism | pantothenate | 1.03 | 0.6936 | 0.973 | 1.063 | 1.10 | 0.7469 | 1.062 | 0.963 |
| Riboflavin Metabolism | riboflavin | 1.03 | 0.3258 | 0.838 | 1.197 | 1.47 | 0.4179 | 1.187 | 0.806 |
| Thiamine Metabolism | thiamin | **0.48** | **0.0486** | **0.571** | **2.022** | 1.26 | 0.8892 | 1.426 | 1.135 |
| Tocopherol Metabolism | alpha-CEHC | 1.14 | 0.7254 | 0.863 | 1.056 | 1.08 | 0.7920 | 0.990 | 0.920 |
|  | gamma-CEHC | **2.84** | **0.0282** | **1.642** | **0.791** | 1.61 | 0.3739 | 1.462 | 0.910 |
|  | gamma-CEHC glucuronide | 1.24 | 0.8717 | 1.1617 | 0.9934 | 1.39 | 0.2712 | 1.2311 | 0.8856 |
|  | alpha-CEHC glucuronide | 0.88 | 0.1810 | 1.2313 | 1.3179 | 1.32 | 0.2891 | 1.4304 | 1.0799 |
|  | alpha-CEHC sulfate | 0.86 | 0.1269 | 1.2080 | 1.2882 | 1.15 | 0.4143 | 1.3264 | 1.1502 |
| Vitamin B-6 Metabolism | pyridoxamine | 1.43 | 0.9757 | 1.340 | 1.084 | 1.08 | 0.9573 | 1.255 | 1.158 |
|  | pyridoxal | 0.82 | 0.1213 | 1.081 | 1.566 | 1.04 | 0.7339 | 1.344 | 1.298 |
|  | pyridoxate | 1.12 | 0.8262 | 1.042 | 1.120 | 1.14 | 0.9793 | 1.143 | 1.003 |
| Inositol Metabolism | chiro-inositol | 2.30 | 0.4885 | 1.428 | 0.952 | 0.72 | 0.6849 | 1.011 | 1.413 |
|  | myo-inositol | 0.88 | 0.2240 | 1.549 | 1.677 | 0.50 | 0.4221 | 1.122 | 2.227 |
| Food Component/Plant | acesulfame | **15691.21** | **0.0049** | **12.324** | **1.790** | 6.38 | 0.9766 | 11.287 | 1.769 |
|  | erythritol | **58.23** | **0.0035** | **47.503** | **0.795** | 0.89 | 0.6037 | 22.8842 | 25.7298 |
|  | saccharin | 0.82 | 0.1737 | 1.546 | 2.065 | 4.02 | 0.2233 | 2.711 | 0.674 |
|  | sucralose | 36.75 | 0.1009 | 1.233 | 1.311 | 2.04 | 0.4038 | 1.645 | 0.806 |

Mean values are scaled intensity in participants with classical PKU (n=5) or variant PKU (n=5) consuming GMP-MF (n=10) and AA-MF (n=10) compared to controls (n=15). Urine was corrected for osmolality. There were no significant differences in urine osmolality between AA-MF and GMP-MF treatments.

^1^Alpha-CEHC, also known as (2,5,7,8-tetramethyl-2-(2'-carboxyethyl)-6-hydroxychroman), is a major water-soluble α-tocopherol metabolite and can be conjugated to other metabolites, such as alpha-CEHC sulfate. Gamma-CEHC, also known as (3-(2,7,8-Trimethyl-3,4-dihydro-2H-chromen-2-yl) propanoate), is a major water-soluble γ-tocopherol metabolite and can be conjugated to other metabolites, such as gamma-CEHC glucuronide. AA-MF, amino acid medical foods; C, classical phenylketonuria; GMP-MF, glycomacropeptide medical foods; PKU, phenylketonuria; V, variant phenylketonuria.

| SUPPLEMENTAL TABLE 4. Laboratory Measures^1, 2^ | | | | | | | | | | | | | | | |
| --- | --- | --- | --- | --- | --- | --- | --- | --- | --- | --- | --- | --- | --- | --- | --- |
|  | AA-MF | | | | |  | GMP-MF | | | | | | | |  |
|  | Means ± SE | | | n | % |  | Means ± SE | | | | | n | | % | *P* |
| Hemoglobin, mg/dL | 14.3 | ± | 0.1 |  |  |  | 14.3 | ± | 0.1 | | |  | |  | 0.75 |
| Below reference range |  |  |  | 1 | 3 |  |  |  |  | | | 1 | | 3 |  |
| Above reference range |  |  |  | 0 | 0 |  |  |  |  | | | 1 | | 3 |  |
| Ferritin, ng/mL^3^ | 47.6 | ± | 9.6 |  |  |  | 51.8 | ± | 11.9 | | |  | |  | 0.36 |
| Below reference range |  |  |  | 1 | 3 |  |  |  |  | | | 2 | | 7 |  |
| Above reference range |  |  |  | 0 | 0 |  |  |  |  | | | 0 | | 0 |  |
| MMA, nmol/L | 154 | ± | 8.9 |  |  |  | 154 | ± | 9.5 | | |  | |  | 1.00 |
| Below reference range |  |  |  | 1 | 3 |  |  |  |  | | | 1 | | 3 |  |
| Above reference range |  |  |  | 0 | 0 |  |  |  |  | | | 1 | | 3 |  |
| Zinc, µg/L^10^ | 82.8 | ± | 3.2 |  |  |  | 78.3 | ± | 1.6 | | |  | |  | 0.14 |
| Below reference range |  |  |  | 1 | 3 |  |  |  |  | | | | 0 | 0 |  |
| Above reference range |  |  |  | 1 | 3 |  |  | | |  |  | | 0 | 0 |  |
| ^1^ Laboratory measures were obtained by fasting venipuncture. Ferritin was measured in the plasma and MMA and zinc were measured in the serum. Statistical analysis included ANOVA with effects for treatment, genotype and genotype treatment interaction, n =30. The *P*-values in this table represent the treatment comparison.  ^2^References ranges include: hemoglobin (mg/dL), 11.6-15.6 (females) & 13.6-17.2 (males); ferritin (ng/mL), 5-204 (females) & 22-275 (males); MMA (nmol/L), 73-271; zinc (µg/dL), 60-120.  ^3^Sample size, n = 21. Participants lacking iron in the MF for one or both treatments were removed.  ^4^One participant was removed due to long-standing history of elevated homocysteine levels, n = 29.  AA-MF, amino acid medical foods; GMP-MF, glycomacropeptide medical foods; MMA, methylmalonic acid. | | | | | | | | | | | | | | | |
